# Spermcast mating with release of zygotes in the small dioecious bivalve *Digitaria digitaria*

**DOI:** 10.1038/s41598-020-69457-2

**Published:** 2020-07-28

**Authors:** Pablo Marina, Javier Urra, Juan de Dios Bueno, José Luis Rueda, Serge Gofas, Carmen Salas

**Affiliations:** 1 Centro Oceanográfico de Málaga, Instituto Español de Oceanografía, Puerto Pesquero s/n, Fuengirola, 29640 Málaga, Spain; 2 Centro de Instrumentación Científica, Universidad de Granada, 18071 Granada, Spain; 3 Departamento de Biología Animal, Facultad de Ciencias, Universidad de Málaga, Campus de Teatinos s/n, 29071 Málaga, Spain

**Keywords:** Zoology, Animal behaviour

## Abstract

*Digitaria digitaria,* a small astartid usually less than 10 mm in length, has a non-brooding behaviour in spite of its limited space for gonad development. This species lives in highly unstable environments with strong currents, which represent a challenge for fertilization and larval settlement. The studied population of *D. digitaria* from the Strait of Gibraltar area was dioecious, with significant predominance of females and sexual dimorphism, where females are larger than males. The reproductive cycle is asynchronous throughout the year, without a resting period, but with successive partial spawning events. The presence of stored sperm in the suprabranchial chamber and inside the gonad of some females, together with the release of eggs along the dorsal axis of both gills, points to internal oocyte fertilization. Bacteriocytes were found in the female and male follicle walls, but no bacteria were observed inside any of the gametes. *Digitaria digitaria* could represent a “missing link” between spermcast mating bivalves with brooded offspring and bivalves with broadcast release of eggs and sperm. The small size, limiting the oocyte production, together with the unstable environment could represent evolutionary pressures towards sperm uptake in *D. digitaria*.

## Introduction

The reproductive patterns of organisms play an essential role in population dynamics, geographical distribution and continuity of the species. The production of gametes, especially that of the eggs, is an energetically expensive activity and therefore is very sensitive to selective pressure^1^. Organisms are capable of taking and assimilating a limited amount of energy from the surrounding environment, and the different ways in which that energy is assigned in order to maximize their fitness, whether growing or reproducing, constitutes a basis of the different life-history strategies of marine invertebrates^2^.

The phylum Mollusca is one of the largest, most diverse and important in the marine environment, where they represent a quarter of the benthic fauna^3^. Sexual behaviour and reproductive patterns are extremely diverse in molluscs^4^. External fertilization in molluscs is frequently associated with dioecious species and a simple type of reproductive behaviour^4^. The release of gametes is usually regulated by the interaction between exogenous factors such as temperature, food availability, salinity or light, and endogenous factors such as neuro-endocrine cycles and genotype^5^. The species with internal fertilization that require several hours of courtship to coordinate the transfer of gametes represent the other extreme^6^. Between both reproductive behaviours there is a wide range of different types of sexual behaviour^7^.

Bivalves with external fertilization are generally medium to large, which allows a large production of gametes that guarantee a high fertilization rate. Conversely, small species (generally < 3 mm in size) usually show offspring protection^8–11^. Sellmer^8^ considered that incubation is an evolutionary adaptation to having a reduced number of young, which in turn is connected to the small parental size; however, brooding is also present in a few large species, such as oysters^12^. Internal fertilization is frequently associated with hermaphroditic species^13,14^. In Bryozoa (with colonies formed by minute zooids), all of the studied species, including stenolaemates and phylactolaemates, have internal intraovarian fertilization of a reduced number of oocytes^15^. For small species of bivalves (between 3 and 10 mm in size), in which the space for the gonads is reduced, with subsequent reduction of the number of eggs, the following question arises: Which sexual behaviour would optimize the reproductive fitness?

The production of female gametes is an expensive energy investment, so such investment must be protected, maximizing the yield through appropriate reproductive strategies. Among those bivalves with different degrees of parental care^9^, hermaphroditism is frequent^16^ although not exclusive. The fertilization in species that incubate their eggs (whether hermaphroditic or not, with most of them small), occurs in the space between demibranchs or elsewhere in the pallial cavity^8,9,17^. On the other hand, some species have eggs that, at the time of egg-laying, are embedded in a gelatinous and adherent mass, which provides an additional protection in the external environment^8,9,18^. Internal cross-fertilization of retained eggs by release, dispersal and uptake of free spermatozoa was named as “spermcast mating”^19^ and the species with this behaviour “spermcast species”. The relationship between small body size, hermaphroditism and egg retention in marine invertebrates would be the evolutionary response to local competition between eggs for fertilization, because body size is the parameter that most strongly influenced the evolution of the fertilization’s mode^14^. When sperm is limited, females of spermcast species are selected to produce fewer but larger eggs that ensure a greater proportion of fertilized eggs^14^. Another hypothesis for the prevalence of brooding in small organisms is the oxygenation of the offspring^11^. According to these authors, if the brooding space is large, the more internal embryos would be smothered and receive insufficient oxygen for development. Environmental factors may also make brooding advantageous e.g. for polychaetes living in vent environments, the high acidification of seawater seems to be behind parental care or lecithotrophic development of the dominant species^20^.

The reproductive cycles are another feature of the reproductive behaviour of the bivalves. They have been particularly analysed in commercial species, such as the mussels^21^ or wedge clams^22^, for fishery management, but there are only a few studies on the reproductive cycle of small or minute species^8,9^. Gametogenic cycles may occur in populations of a species on an annual, semi-annual, or continuous basis. The timing and duration of the gametogenic cycle and the number of cycles within a year may be characteristic of specific populations and it is usually related to environmental variables^9^.

**Figure 1:**
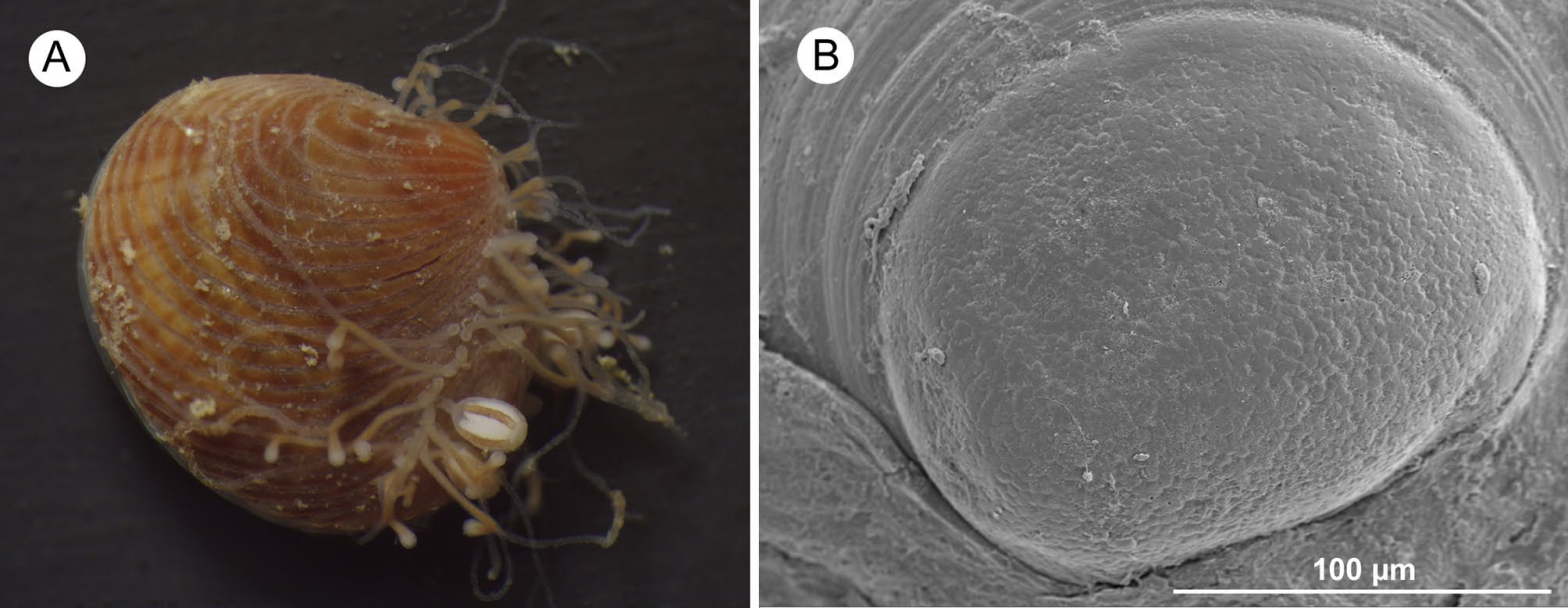
(**A**) Living specimen of *Digitaria digitaria* (length 4.5 mm) with a colony of the hydroid *Monobrachium parasitum* Mereschkowsky, 1877 on the posterior side of the shell. (**B**) SEM view of the large prodissoconch of *D. digitaria*.

*Digitaria digitaria* (Linnaeus, 1758) is a small astartid bivalve, with a size usually not exceeding 10 mm (Fig. 1). It lives in sublittoral bioclastic and sandy bottoms, under the influence of strong currents, from the Bay of Biscay to Guinea and in part of the Mediterranean Sea^23^. The observation of stored spermatozoa in the suprabranchial chamber of some females together with the absence of brooding behaviour in a population living in a very unstable environment with strong currents, such as the Barbate Bay (Strait of Gibraltar area), suggested that this small bivalve may represent an interesting model for the study of the reproductive patterns in Bivalves.

It is unknown whether evolutionary pressure on brooding has been responsible for the origin of sperm uptake, or if spermcast mating arose for different reasons before the adaptation for brooding^13^. According to these authors and in order to clarify the evolutionary context, examples in which brooding does not take place may offer particular insights into the evolution of sperm uptake. In this way the reproductive behaviour of *D. digitaria* would be a “missing link” between spermcast mating bivalves with brooded offspring and broadcast ones with release of eggs and sperm.

## Material and methods

The reproductive behaviour of a population of *D. digitaria* was studied in the Strait of Gibraltar, where strong currents are common due to the entry of surficial Atlantic waters to the Mediterranean basin and the exit of deep Mediterranean outflow waters to the Atlantic basin. Live specimens of *D. digitaria* were collected monthly, from October 1993 to September 1995, in Barbate Bay (36°08′7″N-5°56′7″W) (Atlantic littoral of the Cádiz province), in bioclastic sandy bottoms between 15 and 25 m depth. These samples were collected during a national research project for studying the biodiversity along the Atlantic littoral of Cádiz. The near-bottom seawater temperature was obtained from the project “SeaDataNetFP6” using the model NEMO (*Nucleus for European Modelling of the Ocean*). According to these data, the near-bottom temperature during the study period oscillated between 14 °C in February 1993 and 19 °C in August 1994.

Some additional specimens were collected in bioclastic and sandy bottoms between 15 and 25 m depth in the Mediterranean littoral of Mijas (Málaga province) (36°29′6″N-04°41′30″W). These specimens were used for performing dissections and additional Scanning Electron Microscopy (SEM) and Transmission Electron Microscopy (TEM) analyses. In both instances *D. digitaria* specimens were collected using a small dredge, with a rectangular frame of 42 × 22 cm and 0.5 mm of mesh size.

A total of 2,282 specimens of *D. digitaria* was examined for the study of the reproductive cycle, from which 1589 were used to study monthly changes in biomass (condition index analysis) (ca. 100 by monthly sample) and 693 for the histological study of the gametogenic cycle (ca. 30 by monthly sample). Twenty additional specimens were used for analysing the reproductive behaviour, from which ten specimens were used for dissections, another four for SEM observations, two for performing serially semi-thin sections and four for TEM observations of the male and female gonads, of which semi-thin sections were also obtained. Dissected specimens were photographed using a Nikon DXM camera coupled on a Nikon SMZ100 stereomicroscope.

The condition index (CI) of Beukema^24^, modified by Ansell^25^ was applied:

$${\text{CI }} = {\text{ FDW}}/{\text{L}}^{{3}}$$

where FDW is the flesh dry weight (mg) and L the anterior–posterior length (mm).

For obtaining the flesh dry weight (FDW) the soft parts were dried in an oven at 60 °C for 24 h, and weighed to the nearest milligram. The anterior–posterior length (L) of the shells was measured to the nearest millimetre using a calliper.

**Figure 2:**
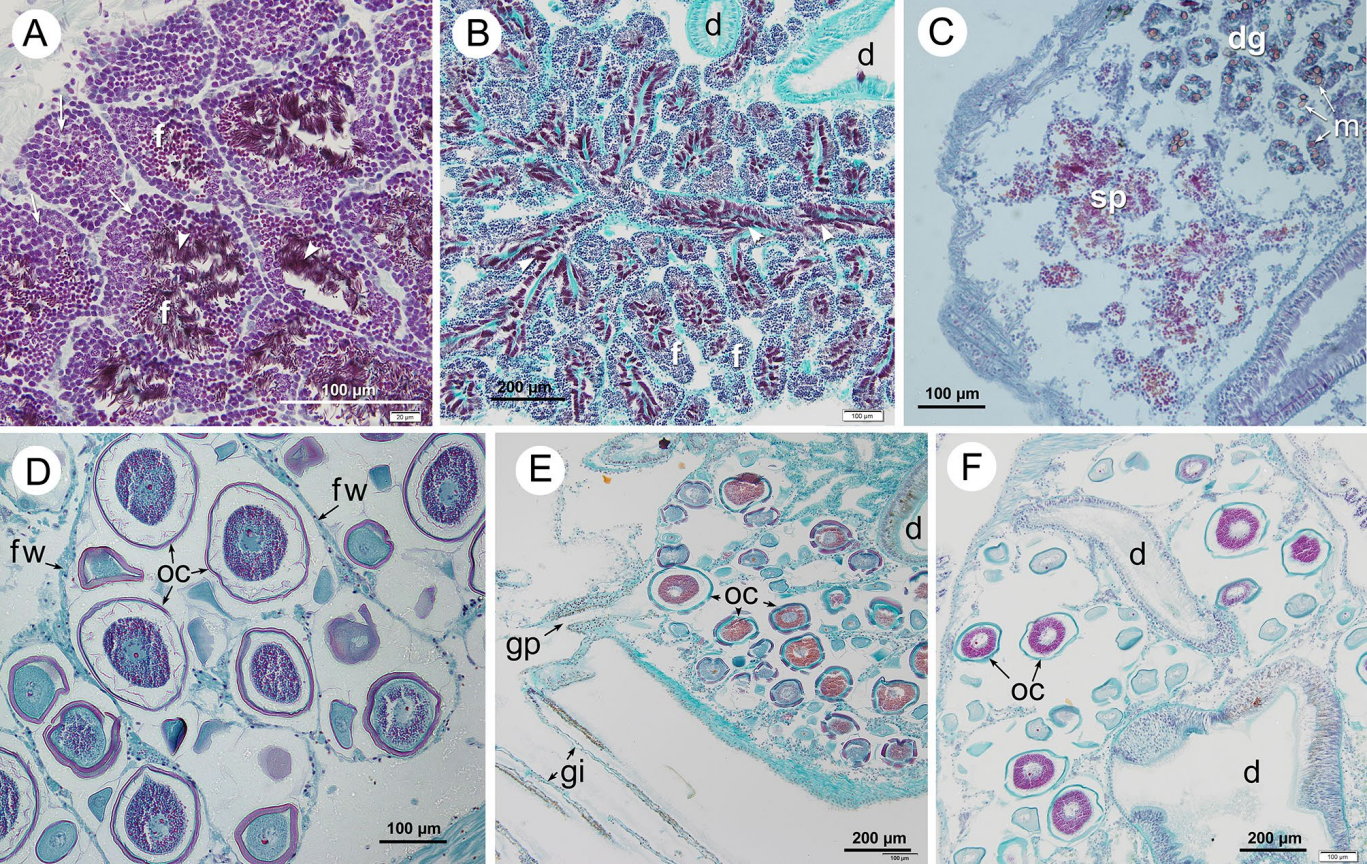
Sagittal sections of different gonadal developmental stages in males (A-C) and females (**D**–**F**) of *D. digitaria*. (**A**) Preactive stage, stained with Hematoxylin–Eosin, the arrows show spermatogonia and the arrowheads spermatozoa. (**B**, **E**) Spawning stage. (**C**–**F**) Postactive stage. (**D**) Active stage. Sections from (**B**) to (**F**) were stained with Hematoxylin-trichromic stain. d, digestive tract; dg, digestive gland; f, follicles; fw, follicle wall; gi, gill; gp, gonadal pore; m, *Marteilia* sp (protozoan parasite of the digestive gland cells); oc, oocyte; sp, spermatozoa.

For histological processing, specimens were fixed in 4% formaldehyde, embedded in paraffin, sectioned at 7 μm and stained with Hematoxylin of Carazzi–eosin, PAS-Alcian Blue or Hematoxylin of Carazzi–trichromic staining (Light Green, Orange G and Acid Fuchsine)^26^. The stages of gonad development were scored according to the scale proposed by De Villiers^27^ as follows: cytolized, pre-active, active, spawning and post-active (Fig. 2).

Samples for Transmission Electron Microscopy (TEM) were fixed in a mixture of 2.5% glutaraldehyde buffered with cacodylate (0.1 M, pH 7.4) for 48 h at 4 °C and post fixed in OsO_4_ (2%) for 2 h at 4 °C, then embedded in epoxy resin Aname EMBED 812 (EMS). Semi-thin sections (0.5–1 μm) were stained with 1% toluidine blue. Ultrathin sections (50–70 nm) were stained with uranyl acetate (1%) followed by lead citrate and examined with a TEM EM 902 ZEISS from the University of Granada or a TEM JEOL-JEM1400 from the University of Málaga. Those specimens embedded in paraffin and partially sectioned used for Scanning Electron Microscopy (SEM) were initially immersed in three 30-min steps in xylene; after this, they were dehydrated by increasing concentrations of ethanol (50%, 70%, 90% and 100%) at room temperature. Each dehydration step took 20 min. They were then critical point CO_2_ dried and sputter-coated with gold. Finally, they were visualized in a SEM JEOL-JSM840 from the University of Málaga.

In general, the sex cannot be distinguished visually if the gonads are not well developed; therefore in those cases sex determination was made microscopically. The Chi squared test was used to check the sex ratio of the studied population based on the percentage of males *vs.* thar of females. The U of Mann Whitney was applied for testing differences between the sizes (shell length and shell height) of females and males. These analyses were done using the software SPSS v.10.

A total of 675 specimens were analysed for calculating the size of sexual maturity. They were grouped into two categories (1) those with developed gonads (active, spawning, post-active or pre-active stage with residual mature gametes) and (2) without developed gonads (undifferentiated or pre-active stage without mature residual gametes in the follicles). Those data were fitted to a logistic regression model (using the software SPSS v.10), from which the size (anterior–posterior length) at which 50% of the individuals had developed gonads (the size of sexual maturity) was estimated.

## Results

### Size of specimens and sex ratio in the population

The shell length of the studied specimens ranged from 1.5 to 9.0 mm, with an average size of 6.3 mm. It was possible to identify the sex of 2,147 specimens, of which 1,031 (48.02%) were male and 1,116 (51.98%) female. Hermaphrodites were not found. Therefore, *D. digitaria* can be considered a dioecious species, with a sex ratio of 1 male: 1.08 females and with a significant higher percentage of females than males (χ^2^ = 3.36 , p < 0.05). In spite of the small difference in the average shell length between males (6.11 ± 1.091 mm) and females (6.33 ± 1.093 mm), and the average shell height between males (5.50 ± 1.016 mm) and females (5.70 ± 1.005 mm), significant differences were also detected, with females displaying larger shell length (U de Mann–Whitney = 514,807, p < 0.01) and height (U de Mann–Whitney = 518,320.5, p < 0.01) than the males.

### Reproductive cycle

**Figure 3:**
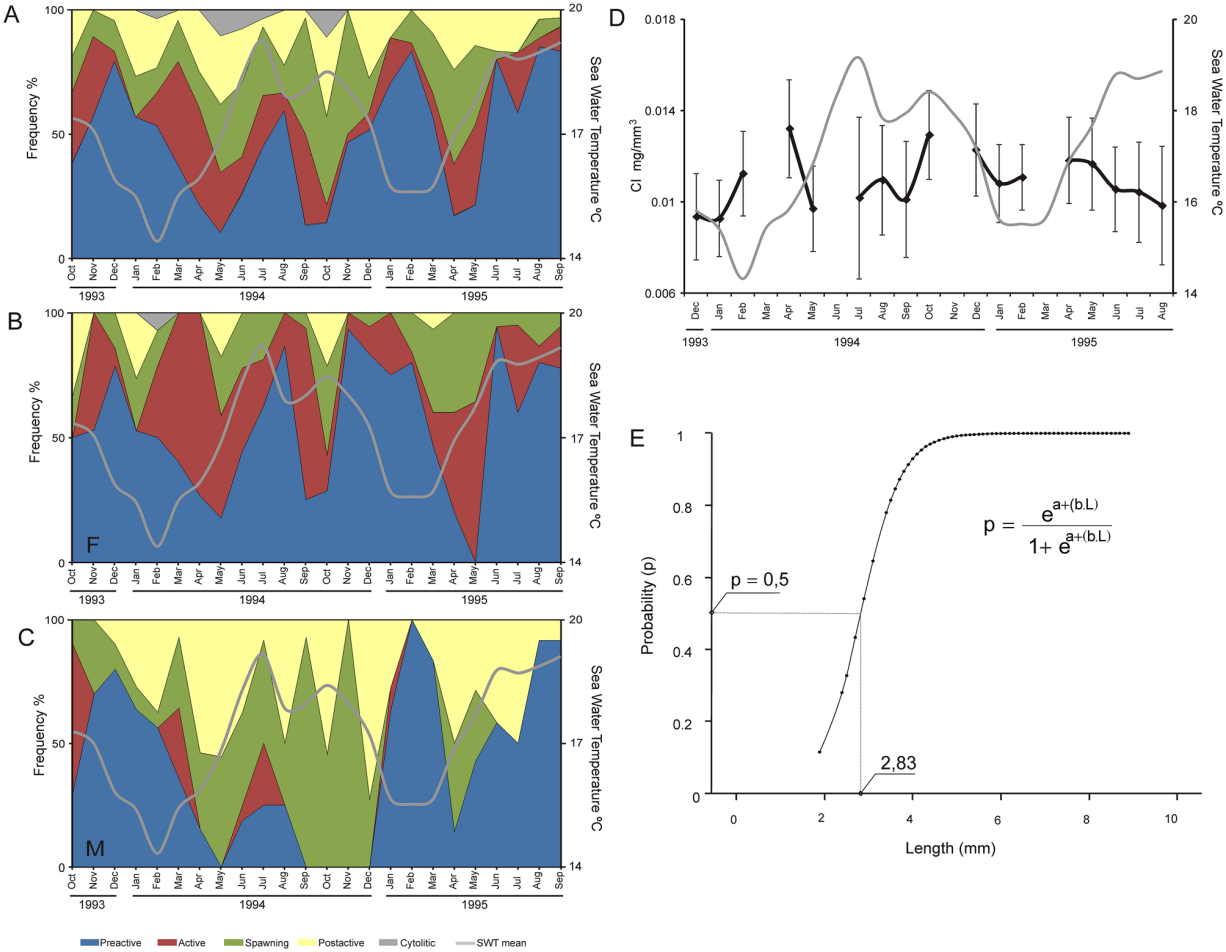
(**A**–**C**) Monthly frequency of the different gonadal development stages of *D. digitaria* and mean seawater temperature (SWT) throughout the study period: (**A**) Whole population. (**B**) Females. (**C**) Males. (**D**) Monthly condition index (CI) of the whole population (black line) and mean seawater temperature (grey line) during the study period. (**E**) Logistic regression line for the analysis of the size of sexual maturity of *D. digitaria* in the studied population. The size of sexual maturity was 2.83 mm, size at which 50% of the population had developed gonads.

The studied population of *Digitaria digitaria* presents functional gonads throughout the year, without a resting period. The gametogenic cycle is asynchronous in both sexes, due to the existence of specimens in three or more stages of gonad development in all monthly samples (Fig. 3A–C). Two main spawning peaks were observed, one in May (28.8% of the population) and another in November (30% of the population) (Fig. 3A). Nevertheless, there were partial spawning events throughout the year. Both peaks occurred when near-bottom seawater temperature was between 17 °C and 18 °C. In females there were two main spawning peaks, the highest one in spring (May) and the lowest one in autumn (October) (Fig. 3B). Remains of mature oocytes were also observed along side new forming ones within the same gonadal follicle, which points to the existence of more than one spawning event per individual during the same year. In males, there were three main sperm release peaks (April, September and November), but partial spawning events were also detected throughout the year (Fig. 3C).

The Condition Index (CI) showed peak values in April and October (when more gonads were in active stage) and lower values in summer and winter months (when more gonads were in postactive stage) (Fig. 3D). No significant correlation was detected between CI and seawater temperature.

The size of sexual maturity in the studied population of *D. digitaria* was reached at 2.83 mm (shell length) (Fig. 3E). Nevertheless, the minimum size with developed gonad was observed in a female of 2.1 mm and in a male of 2.2 mm with spawning and post-active stages respectively.

### Reproductive pattern: gametes release and fertilization

**Figure 4:**
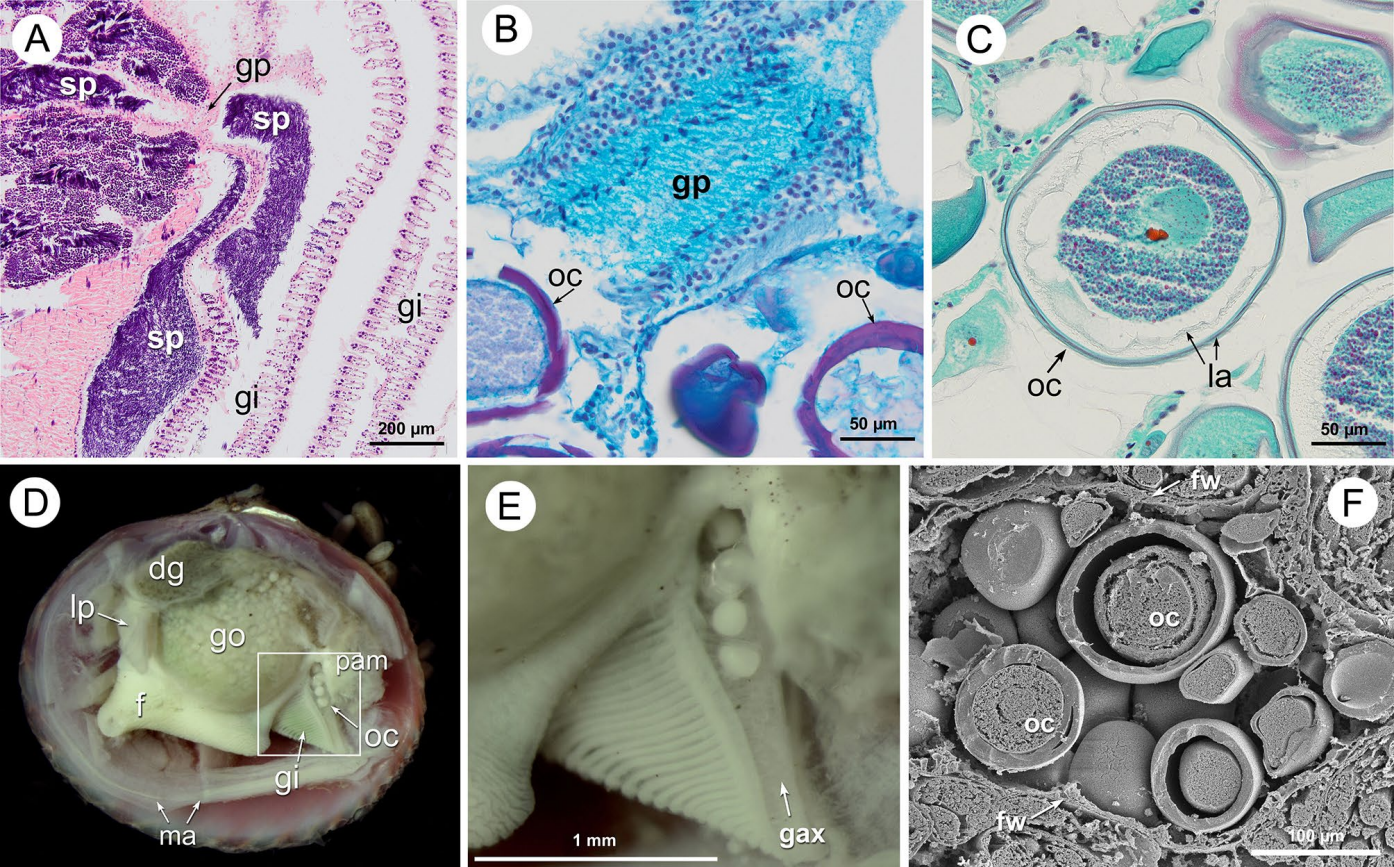
(**A**) Transversal paraffin section stained with Hematoxylin–Eosin showing the release of coalescent sperm. (**B**) Sagittal paraffin section stained with PAS-Alcian blue showing mucous secretion (turquoise colour) in the female gonopore area. (**C**) Sagittal paraffin section stained with Hematoxylin- trichromic stain showing a mature oocyte with several layers. (**D**) Dissection of a female (length 6.3 mm), with left mantle lobe removed, showing release of oocytes on the gill axis. (**E**) Detail of the frame in figure (**D**). (**F**). SEM view of a sectioned female follicle with some oocytes showing different layers. dg, digestive gland; f, foot; fw, follicle wall; gax, gill axis; gi, gill; go, gonad; gp, gonadal pore; la, mucous layers of the oocyte; lp, labial palp; ma, mantle; oc, oocyte; pam, posterior adductor muscle; sp, spermatozoa.

The release of gametes was observed in histological sections in both sexes (Figs. 2B,E, Fig. 4A,B) and by dissection in females (Fig. 4D,E). The genital pores in both sexes are located at the posterior sides of the visceral mass. In females these are located on the dorsal axis of both gills, which form channels through which the eggs are released one by one (Fig. 4D,E). In males, the genital pores open near the insertion of the inner demibranch. Coalescence of the sperm during the spawning has been observed (Fig. 4A). The eggs cast by *D. digitaria* are large (150–180 µm) and they have thick capsular layers (Fig. 4C, F). Mucous secretions by the follicles and at the genital pore area of the females were also observed in histological sections stained with PAS-Alcian blue that stain the mucous secretions with a turquoise colour (Fig. 4B). As the dissections were made on fixed specimens, the mucous secretion embedding the eggs was not apparent.

**Figure 5:**
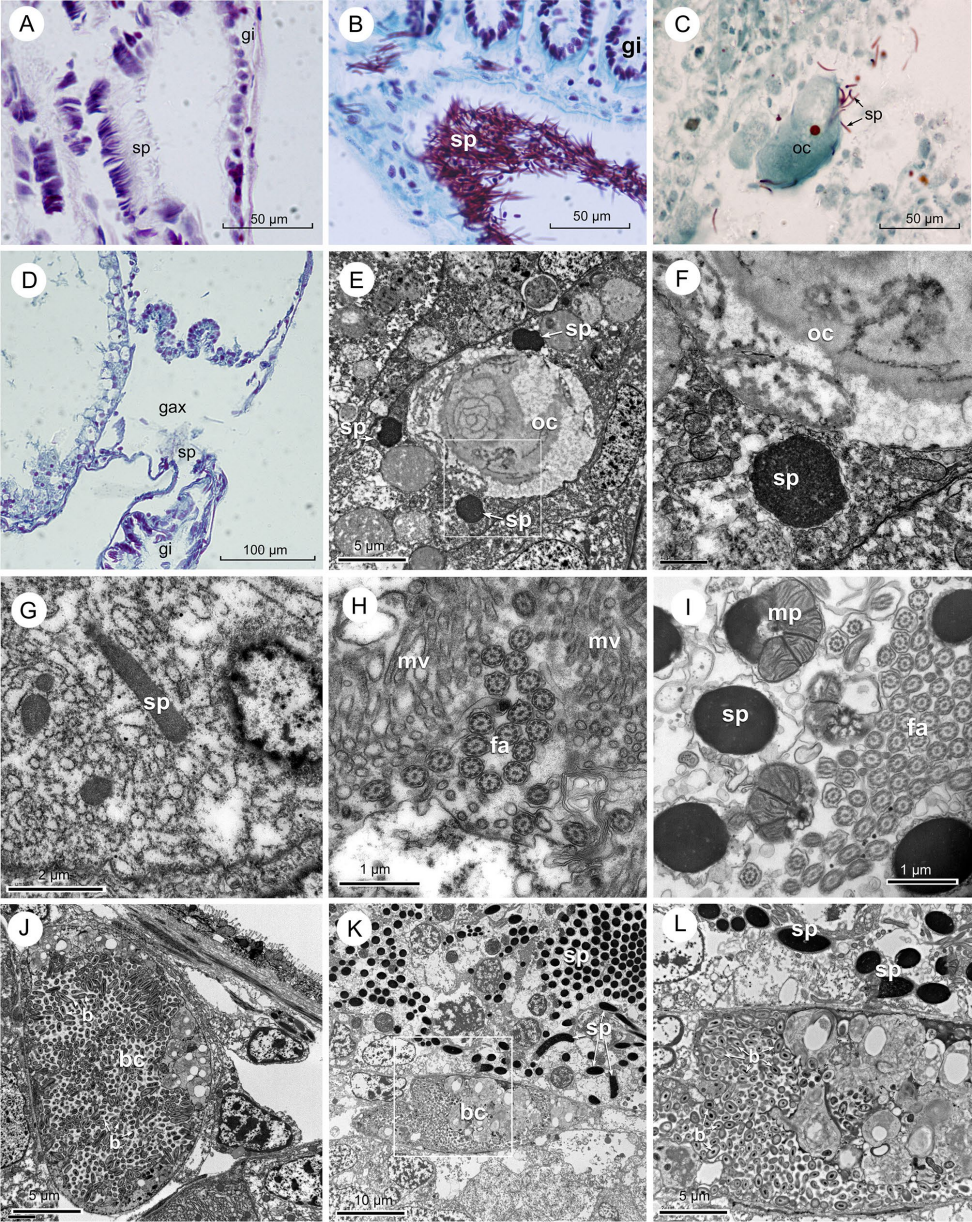
(**A**) Transversal paraffin section, stained with Hematoxylin–Eosin, of the suprabranchial chamber of a gill demibranch with stored spermatozoa. (**B)**Transversal paraffin section of a male spawning stained with Hematoxylin-trichromic, showing sperm between the gill demibranchs. **(C**) Sagittal paraffin sections, stained with Hematoxylin-trichromic stain, showing female follicles with spermatozoa gathering around the oocytes. (**D**) Semithin transversal section, stained with Toluidine blue, showing the aperture between the gill axis and the supra-branchial chamber of the gill. (**E–H)** TEM views of different ultrathin sections of a female gonad with spermatozoa around the oocytes (**E**) Transversal sections showing the nuclei of three spermatozoids around an oocyte. (**F**) Detail of the spermatozoid nucleus from the frame in figure E. (**G**) Longitudinal section of part of a spermatozoid nucleus. (**H**) Transversal sections of spermatozoid flagellar axonemas between the microvilli of two female follicle walls. (**I**). TEM view of a male follicle showing transversal section of the nuclei of several spermatozoids, longitudinal section of two mid-pieces and transversal sections of numerous flagella axonemas. (**J**) Bacteriocyte in the follicle wall of a female. (**K**) Bacteriocyte in the follicle wall of a male. (**L**) Detail of the frame in figure (**H**). *b* bacteria, *bc* bacteriocyte, *gax* gill axis, *gi* gill, *oc* oocyte, *sp* spermatozoa.

Stored sperm was observed in the supra-branchial chamber of some females (Fig. 5A) and is shown for comparison in a spawning male (Fig. 5B). An aperture between the supra-branchial chamber and the gill channel was also detected, from which release of spermatozoa to the gill axis occurs (Fig. 5D). Presence of sperm inside female follicles has been observed in histological paraffin sections (Fig. 5C) and also in TEM ultrathin sections (Fig. 5E–G), where we have observed transversal and longitudinal sections of the sperm nucleus inside some oocytes. Some transversal sections of spermatozoid flagellar axonemas were also observed between the microvillous surface of the follicles (Fig. 5H). For comparison we show a TEM view of a male follicle with different sections of sperm parts (Fig. 5I). In both cases, paraffin and ultrathin sections, the sperm was gathering around the oocytes. Bacteria were also seen inside bacteriocytes located in the follicle walls of the female and male gonads (Fig. 5J–L), but no bacteria could be detected inside the oocytes and spermatozoa.

Taking into account all the above, the fertilization in *D. digitaria* would be internal, within the pallial cavity, along the gill axis channels and also, at least in part, inside the female gonad. The zygotes are released forming a chain along the gill axis (Fig. 4D,E), probably embedded by the mucous secretion produced by the female follicles and gonopores.

## Discussion

*Digitaria digitaria* is a dioecious species, which is the usual pattern in astartids^28^ and in most bivalves^9^. This condition is considered ancestral in molluscs in general, and in bivalves in particular, known in the basal clade of the protobranchs^4^. The bias towards females of the sex-ratio could be related to the small size of the species. Mathematically, the best population growth is expected in female-biased populations^29,30^. Being small in size, *D. digitaria* potentially could be a consecutive hermaphrodite^8,9^. In the studied population, however, males and females of all size classes were found, which indicates that there are no sex changes. The existence of sexual dimorphism is known in extant astartids (*Astarte borealis*, *A. sulcata*, *A. elliptica*)^31,32^ and in American fossil species^33–35^, and in all of them, as in the studied population of *D. digitaria*, females are larger in size than males.

The relatively large eggs of *D. digitaria* (150–180 μm in diameter) are characteristic of a lecithotrophic development^32,36,37^. The latter is supported by the presence of a single and large prodissoconch I (Fig. 1B)^38,39^ without a prodissoconch II denoting a feeding stage. Nevertheless, as in other invertebrates, part of the increased size of eggs of *D. digitaria* appears to be related to the presence of successive layers, some of which are mucosal (“gelatinous”)^40,41^. Larger eggs are fertilized more easily than smaller eggs because they provide larger targets for spermatozoa^42,43^. The latter would be an evolutionary response to local egg competition^14^, overall in an unstable environment with strong currents that favour the dispersion of spermatozoa. The size at first maturity of the analysed population of *D. digitaria* is 2.82 mm, although some smaller specimens with developed gonads were found. This small size represents a disadvantage when producing oocytes, whose number would be conditioned by the available space in the gonad. It is for this reason that some degree of protection of offspring seems to be advantageous^9^.

The extension of the reproductive cycle in bivalves increases as latitude decreases and where temperatures are higher during the winter periods^44,45^. In tropical latitudes most species show extensive reproductive cycles, with functional gonads year round^9^. Another factor which promotes longer reproductive cycles is the abundance of food supply^9^. In the littoral of Málaga, the presence of coastal upwellings favours a high production of phytoplankton, and some bivalves, such as *Callista chione*, a commercial species living in bioclastic sandy bottoms at 20 m depth, maintain functional gonads throughout the year^46^. In Barbate Bay, the near-bottom temperatures are quite stable over the year. In addition, the interaction of the Mediterranean water outflow and the surficial Atlantic waters induces small nutrient-rich upwellings in the Strait of Gibraltar^47^ that would favour a good food supply and the development of gametes throughout the year in *D. digitaria*.

**Figure 6:**
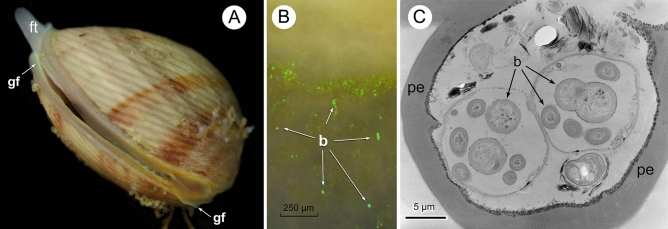
(**A**) Specimen of *D. digitaria* (length 5.4 mm) showing green fluorescence in the mantle edge. (**B**) Culture of fluorescent bacteria from the pitted periostracum of *D. digitaria.* (**C**) Bacteria inside a pit of the periostracum of *D.digitaria*. b, bacteria; ft, foot; gf, green fluorescence in the mantle edge; pe, periostracum.

Although it is difficult to know the number of spawning events in a particular individual, the histological analyses indicate at least two events, because remains of mature gametes have been observed inside follicles in preactive stage, together with new gametes in formation. The asynchronous “pulses” of gametes release throughout the annual cycle, followed by a fast recovery of the gonad, would correspond to a reproductive strategy of small bivalve species optimal for survival. According to^48^, the repetitive spawning in pulses within the same reproductive period is linked to the variability of the environmental and biological conditions for the survival of larvae and juveniles. The handicap of this strategy is that, albeit the chances of one part of the spawning being successful are increased, the possibility of massive success is lost. This reproductive pattern is the one adopted by *D. digitaria* in the studied population, which would fit the “Bet-hedging” reproductive theory^49^, which explains how an individual optimises its suitability in a variable and unpredictable environment. The idea behind such reproductive behaviour is that an individual must decrease the interannual variance of its fitness in order to maximize the long-term adequacy of the population. In fact, the strategy followed by *D. digitaria* could be described as “Conservative Bet-hedging”^50,51^, which is one of the two ways by which an individual can decrease the variance of their adequacy. Thus, the organism would always use the same low-risk reproductive behaviour, producing offspring throughout the reproductive period. In this way, natural selection will ultimately favour those reproductive strategies whose success rate over time is higher, even if their success is lower in one particular moment in time^50,52,53^. Another advantage of the release of gametes in different periods along the year is the decrease of the probability that all oocytes were fertilized by the sperm of just one male. This could increase the genetic variability among the offspring^54^, increasing the probability of adaptation to possible environmental changes and therefore the probability of survival of the species. High levels of multiple paternity have been found in the spermcasting mating freshwater mussel *Margaritifera margartifera*^55^. The wide variability of the shell colour in *D. digitaria* (^23^, see also Figs. 1A and 6A herein) would be an example of the genetic variability present in this species, possibly favoured by this type of reproductive behaviour.

The finding of oocyte release in *D. digitaria* along a grooved structure formed by the dorsal axis of the gills, together with the presence of spermatozoa within the female gonad as well as in the gills, would confirm the internal fertilization followed by release of zygotes. Internal fertilization with storage of sperm is frequent in small brooding bivalves^8, 9^. The sperm can be stored in the gills, e.g. *Mysella tumida*^17^, or in a particular tissue, e.g. *Nutricola tantilla*^56^. Some species with internal fertilization protect the offspring outside the pallial cavity, e.g. *Nucula delphinodonta* which deposits the zygotes in gelatinous sacs attached to the posterior end of the valves, where after 3–4 weeks they are liberated in postlarval stage^8,9^. In *D. digitaria* the eggs could adhere to the substrate through the mucous secretions generated by the female around the genital pore, which would prevent them being washed away by the currents, and give some guarantee that the fertilized eggs would remain in an advantageous environment for their development. Ockelmann^57^ described the existence of eggs with an adherent membrane and a gelatinous covering in other species of astartids (*Astarte borealis, A. montagui, A. crenata, A. elliptica* and *A. sulcata*). All the above observations seem to support the hypothesis that postulates the existence of internal fertilization in species with some type of protection for offspring and/or release of eggs embedded in masses or in gelatinous cords^18^. These authors observed gelatinous egg masses in the lucinid *Phacoides pectinatus* which lives in symbiosis with sulphur-oxidizing bacteria, which furthered the interest to examine the possible link between symbiotic bacteria and the presence of mucilaginous envelope of eggs. In relation to this, a previous study^58^ indicated the presence of bacteria inside the pitted periostracum of *D. digitaria*, which seemed to be spread by the middle mantle fold. The role of these bacteria, and whether they are the same as the ones found in the gonad, is still unknown. The presence of bacteriocytes on the female and male gonads suggests the existence of symbiotic bacteria with a vertical transmission^59^.

Under light, the mantle edge of *D. digitaria* displays green fluorescence (Fig. 6A), and preliminary essays of culture of the bacteria from the periostracum (Fig. 6C) have shown the presence of some fluorescent colonies (Fig. 6B). The identification of the symbiotic bacteria is still under study and the effects for the bivalve are still unknown. Nevertheless, the presence of bacteriocytes in the male gonad was previously unknown in marine bivalves, in which the vertical transmission was only observed in oocytes of chemosymbiotic species^60,61^. The release of gametes within a mucous secretion forming a compact sperm mass or a chain of eggs could favour the bacterial transmission into the offspring. All these features would suggest a possible function of the bacteria in the reproductive pattern; one possibility would be to attract the spermatozoa to females in a very unstable environment. Some studies have pointed to the positive phototaxy of zooxanthellae toward the green fluorescence of some cnidarians, which is used by the latter for capturing of the symbionts or as prey attractant^62–64^.

## Conclusions

The small bivalve *D. digitaria*, living in an unstable but nutrient-rich environment, shows a continuous reproductive cycle with functional gonads in both sexes throughout the year, with frequent partial spawning events and without resting period.

The females receive the sperm from the inhalant water current, being stored in the suprabranchial chamber, from which they fertilize the oocytes released along the gill axis. Spermatozoids inside the ovary were observed. All the latter indicate internal fertilization, with release of zygotes attached to the bottom by their mucous envelope.

The reproductive behaviour of *D. digitaria*, in which brooding does not occur, would be a “missing link” between spermcast mating bivalves with brooded offspring and broadcast ones with eggs and sperm release.

The small size of *D. digitaria*, limiting the production of oocytes, together with the unstable environment where it lives seem to be the evolutionary pressures and challenge for the sperm uptake in this small astartid.

## Data Availability

The raw data used in this work are available in the Dryad repository under 10.5061/dryad.t1g1jwszd.

## References

[1] Hayward A, Gillooly JF (2011). The cost of sex: quantifying energetic investment in gamete production by males and females. PLoS ONE.

[2] Llodra ER (2002). Fecundity and life-history strategies in marine invertebrates. Adv. Mar. Biol..

[3] Appeltans W (2002). The magnitude of global marine species diversity. Curr. Biol..

[4] Collin R (2013). Phylogenetic patterns and phenotypic plasticity of molluscan sexual systems. Integr. Comp. Biol..

[5] Giese AC, Pearse JS (1977). Reproduction of Marine Invertebrates Vol. 5. Molluscs: Pelecypods and Lesser Classes.

[6] Runham NW, Adiyodi KG, Adiyodi RG (1992). Mollusca. Reproductive Biology of Invertebrates, Vol. 5. Sexual Differentiation and Behavior.

[7] Leonard JL (2013). Williams' paradox and the role of phenotypic plasticity in sexual systems. Integr. Comp. Biol..

[8] Sellmer G (1967). Functional morphology and ecological life history of the gem clam *Gemma gemma* (Eulamellibranchia: Veneridae). Malacologia.

[9] Sastry AN, Giese AC, Pearse JS (1979). Pelecypoda (excluding Ostreidae). Reproduction of Marine Invertebrates, Vol. 5. Molluscs: Pelecypods and Lesser Classes.

[10] Strathmann RR, Strathmann MF (1982). The relationship between adult size and brooding in marine invertebrates. Am. Nat..

[11] Strathmann RR, Strathmann MF, Emson RH (1984). Does limited brood capacity link adult size, brooding, and simultaneous hermaphroditism? A test with the starfish *Asterina phylactica*. Am. Nat..

[12] O’Foighil D, Taylor DJ (2000). Evolution of parental care and brooding in oysters. Mol. Phylogenet. Evol..

[13] Bishop JDD, Pemberton AJ (2006). The third way: spermcast mating in sessile marine invertebrates. Integr. Comp. Biol..

[14] Henshaw JM, Marshall DJ, Jennions MD, Kokko H (2014). Local gamete competition explains sex allocation and fertilization strategies in the sea. Am. Nat..

[15] Ostrovsky AN (2013). Evolution of Sexual Reproduction in Marine Invertebrates: Example of Gymnolaemate Bryozoans.

[16] Pelseneer, P. Les lamellibranches de l’expedition du Siboga. *Siboga Expeditie, Livre 61, Monographie* 53a, 1–125 (1911).

[17] O’Foighil D (1985). Sperm transfer and storage in the brooding bivalve *Mysella tumida*. Biol. Bull..

[18] Collin R, Giribet G (2010). Report of a cohesive gelatinous egg mass produced by a tropical marine bivalve. Invert. Biol..

[19] Pemberton AJ, Hughes RN, Manrìquez PH, Bishop JD (2003). Efficient utilization of very dilute aquatic sperm: sperm competition may be more likely than sperm limitation when eggs are retained. Proc. R. Soc. B.

[20] Lucey NM (2015). To brood or not to brood: Are marine invertebrates that protect their offspring more resilient to ocean acidification?. Sci. Rep..

[21] Gosling, E. *The Mussel* Mytilus*: Ecology, Physiology, Genetics and Culture,* xiii + 589 pp (Elsevier, 1992).

[22] Tirado C, Salas C (1998). Reproduction and fecundity of *Donax trunculus* L., 1758 (Bivalvia: Donacidae) in the littoral of Málaga (southern Spain). J. Shellfish Res..

[23] Gofas, S., Salas, C. & Moreno, D. (eds.) *Moluscos Marinos de Andalucía*, Vol. 1 and 2 (Universidad de Málaga, 2011).

[24] Beukema JJ (1974). Seasonal changes in the biomass of the macro-benthos of a tidal flat area in the Dutch Wadden Sea. Neth. J. Sea Res..

[25] Ansell AD, Frenkiel L, Moueza M (1980). Seasonal changes in tissue weight and biochemical composition for the bivalve *Donax trunculus* L. on the Algerian coast. J. Exp. Mar. Biol. Ecol..

[26] Gutierrez M (1967). Coloración histológica para ovarios de peces, crustáceos y moluscos. Invest. Pesq..

[27] De Villiers G (1975). Reproduction of the sand mussel *Donax serra* Röding. Investig. Rep. Sea Fish. Brch S. Afr..

[28] Mikkelsen PM, Bieler R (2008). Seashells of Southern Florida: Living Marine Mollusks of the Florida Keys and Adjacent Regions. Bivalves.

[29] Karlin S, Lessard S (1983). On the optimal sex ratio. Proc. Natl. Acad. Sci. USA.

[30] Vlad MO (1989). The optimal sex ratio for age-structured populations. Math. Biosci..

[31] Saleuddin ASM (1964). The gonads and reproductive cycle of *Astarte sulcata* (Da Costa) and Sexuality in *A. elliptica* (Brown). Proc. Malac. Soc. Lond..

[32] Ockelmann KW (1965). Developmental types in marine bivalves and their distribution along the Atlantic coast of Europe.

[33] Heaslip WG, Westermann GEG (1969). Sexual dimorphism in bivalves. Sexual Dimorphism in Fossil Metazoa and Taxonomic Implications.

[34] Kauffman EG, Buddenhagen CHP, Westermann GEG (1969). Sexual Dimorphism in Paleocene *Astarte* (Bivalvia) of Maryland. Sexual Dimorphism in Fossil Metazoa and Taxonomic Implications.

[35] Kelley PH (1980). Sexual dimorphism in Miocene Coastal Plain *Astarte* populations. J. Miss. Acad. Sci..

[36] Thorson G (1950). Reproduction and larval ecology of marine bottom invertebrates. Biol. Rev. Camb. Philos. Soc..

[37] O’Foighil D (1989). Role of spermatozeugmata in the spawning ecology of the brooding oyster *Ostrea edulis*. Gamete Res..

[38] Salas C, Gofas S (1997). Brooding and non-brooding *Dacrydium* (Bivalvia: Mytilidae): a review of the Atlantic species. J. Moll. Stud..

[39] Linse K, Page TJ (2003). Evidence of brooding in Southern Ocean limid bivalves. J. Moll. Stud..

[40] Podolsky RD (2001). Evolution of egg target size: an analysis of selection on correlated characters. Evolution.

[41] Podolsky RD (2004). Life history consequences of investment in free spawned eggs and their accessory coats. Am. Nat..

[42] Levitan DR (1993). The importance of sperm limitation to the evolution of egg size in marine invertebrates. Am. Nat..

[43] Crean AJ, Marshall DJ (2008). Gamete plasticity in a broadcast spawning marine invertebrate. Proc. Natl. Acad. Sci. USA.

[44] Giese AC (1959). Comparative physiology: annual reproductive cycles of marine invertebrates. Ann. Rev. Physiol..

[45] Mac Donald BA, Thompson RJ (1988). Intraspecific variation in growth and reproduction in latitudinally differentiated populations of the giant scallop *Placopecten magellanicus* (Gmelin). Biol. Bull..

[46] Tirado C, Salas C, López JI (2002). Reproduction of *Callista chione* L. 1758 (Bivalvia: Veneridae) in the littoral of Málaga (southern Spain). J. Shellfish Res..

[47] Millot C (2009). Another description of the Mediterranean Sea outflow. Progr. Oceanogr..

[48] Olive PJ (1985). Physiological adaptations and the concepts of optimal reproductive strategy and physiological constraint in marine invertebrates. Symp. Soc. Exper. Biol..

[49] Slatkin M (1974). Hedging one’s evolutionary bets. Nature.

[50] Seger J, Brockman HJ, Harvey PH, Partridge L (1988). What is bet-hedging?. Oxford Surveys in Evolutionary Biology.

[51] Philippi T, Seger J (1989). Hedging one’s evolutionary bets, revisited. Trends Ecol. Evol..

[52] King OD, Masel J (2007). The evolution of bet-hedging adaptations to rare scenarios. Theor. Popul. Biol..

[53] Olofsson H, Ripa J, Jonzen N (2009). Bet-hedging as an evolutionary game: the trade-off between egg size and number. Proc. R. Soc. B.

[54] Fox CW, Rauter CM (2003). Bet-hedging and the evolution of multiple mating. Evol. Ecol. Res..

[55] Wacker S, Larsen BM, Jakobsen P, Karlsson S (2018). High levels of multiple paternity in a spermcast mating freshwater mussel. Ecol. Evol..

[56] Lützen J, Jespersen Å, Russell MP (2015). The Pacific clam *Nutricola tantilla* (Bivalvia: Veneridae) has separate sexes and makes use of brood protection and sperm storage. J. Moll. Stud..

[57] Ockelmann KW (1958). The zoology of East Greenland Marine Lamellibranchiata. Medddelelser om Grönland.

[58] Salas C, Marina P, Checa AG, Rueda JL (2012). The periostracum of *Digitaria digitaria* (Bivalvia: Astartidae): formation and structure. J. Moll. Stud..

[59] Bright M, Bulgheresi S (2010). A complex journey: transmission of microbial symbionts. Nat. Rev. Microbiol..

[60] Ikuta T (2016). Surfing the vegetal pole in a small population: extracellular vertical transmission of an 'intracellular' deep-sea clam symbiont. R. Soc. Op. Sci..

[61] Russell SL, McCartney E, Cavanaugh CM (2018). Transmission strategies in a chemosynthetic symbiosis: detection and quantification of symbionts in host tissues and their environment. Proc. R. Soc. B.

[62] Hollingsworth LL, Kinzie RA, Lewis TD, Krupp DA, Leong JAC (2005). Phototaxis of motile zooxanthellae to green light may facilitate symbiont capture by coral larvae. Coral Reefs.

[63] Haddock SHD, Dunn CW (2015). Fluorescent proteins function as a prey attractant: Experimental evidence from the hydromedusa *Olindias formosus* and other marine organisms. Biol. Open.

[64] Aihara Y, Maruyama S, Baird AH, Iguchi A, Takahashi S, Minagawa J (2019). Green fluorescence from cnidarian hosts attracts symbiotic algae. Proc. Natl. Acad. Sci. USA.

